# Vanillin Prevents Doxorubicin-Induced Apoptosis and Oxidative Stress in Rat H9c2 Cardiomyocytes

**DOI:** 10.3390/nu12082317

**Published:** 2020-08-01

**Authors:** Ivana Sirangelo, Luigi Sapio, Angela Ragone, Silvio Naviglio, Clara Iannuzzi, Daniela Barone, Antonio Giordano, Margherita Borriello

**Affiliations:** 1 Department of Precision Medicine, Università degli Studi della Campania “Luigi Vanvitelli”, Via L. De Crecchio 7, 80138 Naples, Italy; luigi.sapio@unicampania.it (L.S.); angela.ragone@unicampania.it (A.R.); silvio.naviglio@unicampania.it (S.N.); clara.iannuzzi@unicampania.it (C.I.); margherita.borriello@unicampania.it (M.B.); 2 Cell Biology and Biotherapy Unit, Istituto Nazionale Tumori—IRCCS—Fondazione G. Pascale, 80131 Napoli, Italy; d.barone@istitutotumori.na.it; 3 Sbarro Institute for Cancer Research and Molecular Medicine, Center for Biotechnology, College of Science and Technology, Temple University, Philadelphia, PA 19122, USA; giordano@temple.edu; 4 Department of Medical Biotechnology, University of Siena, 53100 Siena, Italy

**Keywords:** doxorubicin, ROS, vanillin, H9c2 cells, chemopreventive agents, antioxidant, cardiotoxicity

## Abstract

Doxorubicin (doxo) is an effective anticancer compound in several tumor types. However, as a consequence of oxidative stress induction and ROS overproduction, its high cardiotoxicity demands urgent attention. Vanillin possesses antioxidant, antiproliferative, antidepressant and anti-glycating properties. Therefore, we investigated the potential vanillin protective effects against doxo-induced cardiotoxicity in H9c2 cells. Using multiparametric approach, we demonstrated that vanillin restored both cell viability and damage in response to doxo exposure. Contextually, vanillin decreased sub-G1 appearance and caspase-3 and PARP1 activation, reducing the doxo-related apoptosis induction. From a mechanistic point of view, vanillin hindered doxo-induced ROS accumulation and impaired the ERK phosphorylation. Notably, besides the cardioprotective effects, vanillin did not counteract the doxo effectiveness in osteosarcoma cells. Taken together, our results suggest that vanillin ameliorates doxo-induced toxicity in H9c2 cells, opening new avenues for developing alternative therapeutic approaches to prevent the anthracycline-related cardiotoxicity and to improve the long-term outcome of antineoplastic treatment.

## 1. Introduction

Classified as an anthracycline antibiotic, doxorubicin (doxo) is an effective anti-neoplastic drug in a broad range of cancers, including breast cancer, leukemia, lymphoma and other solid tumors [1,2]. Nevertheless, due to its pervasive cardiotoxic effects, long-term doxo administration is usually not recommended in cancer patients. Indeed, despite the beneficial effects in tumor progression, doxo-mediated cardiovascular diseases are quite frequent in long-term cancer survivors, where they may significantly contribute to heart failure and death [3,4]. To date, several mechanisms have been implicated in the doxo-induced cardiotoxicity, such as increased oxidative stress and lipid peroxidation, DNA damage, apoptosis and, more recently, autophagy [5]. However, increased reactive oxygen species (ROS) production remains the major mechanism associated with doxo-induced oxidative stress, mitochondria injury and cellular apoptosis in cardiac cells [6,7,8,9]. The restricted antioxidant enzymes pool, especially catalases, in cardiac cells are often inadequate to counteract the ROS production amount during doxo administration [10,11].

In order to reduce the doxo-related cardiac injuries, several approaches have been proposed, including the usage of new doxo formulations, less toxic, but always effective against cancer cells and cardioprotective supplemental therapies. Currently, the employment of additional cardioprotective agents is strongly recommended as coadjutant procedure in alkylating-based treatment [12,13,14]. In this respect, significant efforts have been accomplished in order to identify novel and more effective adjuvant agents against doxo-induced cardiotoxicity. Several molecules, such as beta blockers, angiotensin receptor blockers, dexrazoxane, have also been explored in preclinical studies [15,16,17]. However, a concomitant reduction in anti-cancer efficacy and side effects intensification have radically hampered the following clinical trials investigation [18,19]. Recently, due to their well-known antioxidant properties, many attentions have been paid to the plant-derived polyphenols as a possible strategy in the prevention of doxo-induced cardiotoxicity [5,20,21]. Speaking of which, it has been reported that curcumin, a natural phenol abundant in turmeric, is able to ameliorate the doxo-induced cardiotoxic effect both in vitro and in vivo studies [22,23,24]. The mechanisms by which curcumin acts as cardioprotective compound seems to be related to the attenuation of oxidative stress, inflammation and associated cell death pathways [23,25,26,27,28]. However, it should be clarified that only upon curcumin pre-treatment a protective effect has been observed, whereas concomitant curcumin-doxo treatment potentiated the cardiotoxicity [28,29]. Despite the multiple evidence in support of curcumin efficacy and safety, its application as a potential chemotherapeutic and/or cardioprotective agent is strongly hindered by its poor bioavailability and low aqueous solubility [30,31]. At physiological pH, curcumin is rapidly hydrolyzed and, even after high-doses administration, only minimal fluctuations have been revealed in in vivo models [32,33]. With the purpose of identifying the agents responsible for the beneficial curcumin properties, the analysis of the curcumin-related degradation products has recently received more attention, especially in the oncological field [34,35]. Among the others, vanillin, the major component of vanilla bean extract, is one of the main and stable degradation products of curcumin [34,35,36]. Vanillin is a widely known antioxidative, anti-apoptotic, anti-inflammatory, neuroprotective and anticancer compound [37,38,39]. However, no data are currently available concerning the vanillin-mediated cardioprotective features. For this reason, and taking into account all the mentioned perspectives, the present study has been designed to assess the consequences of vanillin treatment on doxo-induced cardiotoxicity. Using a broadly cardiomyocytes in vitro model, and employing a multiparametric approach, we determined the consequences of vanillin administration in H9c2 cells both as single agent and in combination with doxo. Additionally, with the intention of supporting the usage of vanillin as an effective antagonist just only toward cardiotoxicity and not against doxo efficacy in cancer cell, an identical experimental strategy has also been applied to a representative osteosarcoma model, an aggressive tumor in which doxo still represents one of the only effective weapons [40].

## 2. Materials and Methods

### 2.1. Materials

Doxorubicin (D1515), vanillin (V1104), 3-(4,5-dimethylthiazol-2-yl)-2,5-diphenyl-tetrazolium bromide (MTT) (Sigma-Aldrich Co., St. Louis, MO, USA). Antibodies: anti-Cleaved Caspase-3 (Asp175) (5A1E) (#9664); anti-p44/42 MAPK (ERK1/2) (#9102), anti-phospho-p44/42 MAPK (ERK1/2) (Thr202/Tyr204) (#9101) (Cell Signaling Technology). anti-PARP (#P7605), anti-PARP (Cleaved-Asp214) (#SAB4500487), anti-β-actin AC-74 (#A2228) (Sigma-Aldrich). anti-α-tubulin (B-7) (sc-5286) (Santa Cruz Biotechnology). Secondary antibodies: goat anti-rabbit (GtxRb-003-DHRPX) and goat anti-mouse (GtxMu-003-EHRPX.0.05) (Immunoreagents, Inc.).

### 2.2. Cell Cultures and Treatments

Embryonic rat cardiac tissue-derived H9c2 cardiomyoblasts (ATCC^®^ CRL-1446) and human U2OS osteosarcoma cells (ATCC^®^ CRL-1446) were cultured in Dulbecco’s minimum essential medium (DMEM) (AL007, Microgem, Naples, Italy) supplemented with 10% FBS, 2.0-mM glutamine (X0550, Microgem), 100 units/mL penicillin and 100-mg/mL streptomycin (A001, HiMedia) in a 5.0% CO_2_ humidified environment at 37 °C. Cells were grown for 18 h before starting treatments. The experimental groups were: UT: untreated cells; doxo: cells treated with doxo 20-μM for 24 h; Pretr: cells pretreated with 100-μM vanillin for 18 h and then treated with doxo 20-μM for 24 h; Cotr: cells treated in parallel with vanillin 100-μM and doxo 20-μM for 24 h; Van: cells treated with vanillin 100 μM.

### 2.3. MTT Assay

Cell viability was assessed through the ability of cells to reduce the metabolic dye 3-[4-dimethylthiazol-2-yl]-2,5-diphenyltetrazolium bromide (MTT) to a blue formazan product. After treatments, cells were rinsed with phosphate buffer solution (PBS). A stock solution of MTT (5-mg/mL in PBS) was diluted ten times in cell medium and incubated for 3 h at 37 °C. After removing the medium, cells were treated with isopropyl alcohol, 0.1-M HCl for 20 min. Levels of reduced MTT were assayed by measuring the difference in absorbance between 570 and 690 nm. Data are expressed, as percentage reduction of MTT with respect to the control ± S.D. Data were obtained from five independent experiments carried out in triplicate.

### 2.4. Evaluation of LDH, AST and ALT Activities

The activity of lactate dehydrogenase, (LDH), aspartate aminotransferase (AST) and alanine aminotransferase (ALT) intracellular enzymes was evaluated in the media of each experimental groups, using the Abbott Lab Chemistry Analyzer ci 8200. Relative enzyme activity was determined in accordance with the manufacturer’s instructions.

### 2.5. Trypan Blue Assay

To determine the number of dead cells in the experimental groups, Trypan Blue assay was employed. Trypan Blu is a cell membrane-impermeable dye and, therefore, its presence inside the cells is due to damaged membranes. Upon the entry into the cells, Trypan Blue renders the cells dark blue. Briefly, cells were seeded in 10% FBS-containing medium in a 6-well plate at a density of 1 × 10^5^ cell/well for 18 h at 37 °C and then treated as above described. After 24 h of treatment, cells were collected and cell counting was performed by mixing 10 μL of cell suspension with an equal volume of Trypan Blue (0.4%, *v/v*). The number of blue stained cells (dead) was recorded. For U2OS experiments, besides the number of blue stained cells (dead cells), also not-stained cells (viable cells) were counted. Trypan Blue experiments were performed three times (in replicates of six wells for each data point in each experiment). Data are presented as means ± standard deviation for a representative experiment.

### 2.6. DAPI Nuclear Staining

Changes in nuclear morphology were evaluated using DAPI fluorescent dye. After treatments, H9c2 cells were fixed with 3% paraformaldehyde and permeabilized with 0.1% Triton X-100 before the incubation with Hoechst at the final concentration of 1μg/mL for 10 min. Then, the cells were washed three times with PBS and observed under fluorescent microscope (Leica). The fluorescence intensity was quantified using Image J software.

### 2.7. Propidium Iodide-Cell Staining

After treatments, 2,5 × 10^5^ cells were collected and resuspended in 500 μL of 50-μg/mL propidium iodide (PI) solution and incubated in the dark for 30 min. After this time, samples were analyzed by acquired on a FACS-Calibur flow cytometer using the Cell Quest software (Becton Dickinson, Franklin Lakes, NJ, USA). The analysis of sub-G1 variation was performed using ModFitLT version 3 software (Verity).

### 2.8. Western Blotting Analysis

Pelleted cells were lysed in 3–5 volume of RIPA Buffer (R0278, Sigma-Aldrich), containing Protease Inhibitor Cocktail (P8340, Sigma-Aldrich) and Phosphatase Inhibitor Cocktail (P2850, Sigma-Aldrich). After centrifugation, supernatant phase was collected and underwent to protein content quantification by Bradford Assay (39222.02, Serva, Rome, Italy). An equal protein amount from each sample was diluted in Laemmli buffer 4X (S3401, Sigma-Aldrich), and finally boiled 5 min at 95 °C. Successively, polyacrylamide gels (10687.02, Serva) were loaded with 20–40 μg of whole extracts and SDS-PAGE was started. Using Mini Trans-Blot (Bio-Rad Laboratories), separated proteins were moved to nitrocellulose membranes (GE10600008 Sigma-Aldrich), which were finally blocked one hour in no-fat milk 5% *w*/*v* to prevent nonspecific antibody bindings. After overnight incubation in primary antibody, membranes were washed in TBS Tween-20 (TC287, HIMEDIA) three times prior to and following conjugated secondary antibody ligation. Horseradish Peroxidase-mediated light reaction was made by enhanced chemiluminescence detection kit (EuroClone) and detected with Chemi Doc XRS (Bio-Rad).

### 2.9. Detection of Intracellular ROS

Intracellular ROS were detected by means of an oxidation-sensitive fluorescent probe 2′,7′-dichlorofluorescin diacetate (DCFH-DA) as previously reported [41]. Cells were grown in 12-well plates (2.5 × 10^6^ cell/well), pre-incubated with DCFH-DA for 30 min and then incubated with protein samples for 24 h. Control experiments were performed using untreated cells and cells exposed to a 0.001-M H_2_O_2_. After incubation, cells were washed twice with PBS buffer and then lysed with Tris-HCl 0.5 M, pH 7.6, 1% SDS. The non-fluorescent DCFH-DA is converted, by oxidation, to the fluorescent molecule 2′,7′-dichlorofluorescein (DCF). DCF fluorescence intensity was quantified on a PerkinElmer Life Sciences LS 55 spectrofluorometer using an excitation wavelength of 488 nm and an emission wavelength of 530 nm. Data are expressed as average ±S.D. from five independent experiments carried out in triplicate.

### 2.10. Statistical Analysis

Experimental results were subjected to rigorous statistical analysis. In details, student’s *t*-distribution and one-way analysis of variance (ANOVA) were employed to calculate the significance in means between treated vs untreated groups and among different experimental groups, respectively. *p*-values of less than 0.05 were recognized as significant and indicated with graphic star as follows: *: *p* < 0.05; **: *p* < 0.01; *** *p* ˂ 0.001. All densitometric analyses were performed by Image J (NIH, Bethesda).

## 3. Results

### 3.1. Vanillin Strongly Reduces Doxo-Induced Toxicity in H9C2 Cells

**Figure 1:**
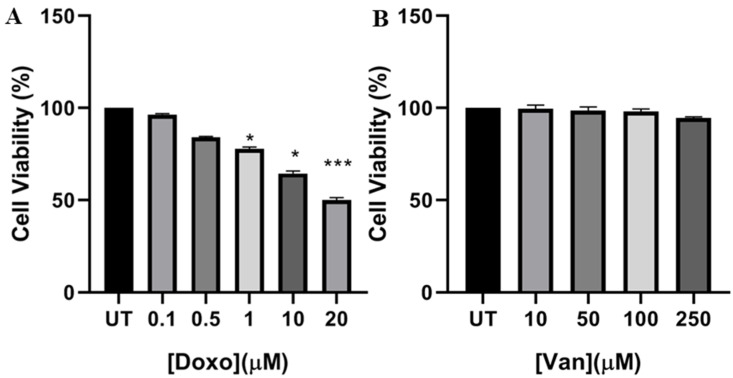
Effect of doxo and vanillin on cell viability. Cell viability was evaluated by MTT assay in H9c2 cells exposed for 24 h to (**A**) increasing concentration of doxo (from 0.1 to 20 μM) and to (**B**) different concentrations of vanillin (20, 50, 100 and 150 μM) UT—untreated cells. Data are expressed as average percentage of MTT reduction ± SD relative to untreated cells from triplicate wells from 5 separate experiments. *: *p* < 0.05; ***: *p* < 0.001 by unpaired *t*-test. Other experimental details are described in Materials and Methods section.

In order to evaluate the potential vanillin cardioprotective properties in response to doxo treatment, first, we assessed the relative doxo-induced cytotoxicity in our experimental settings. According to previous findings, H9c2 cardiac cells were exposed for 24 h to increasing concentration of doxo (from 0.1 up to 20 μM) and then cell viability was evaluated by MTT assay [28]. As shown in Figure 1A, up to 0.5-μM doxo no significant changes were detected in cell viability, whereas a reduction of 20% and 35% was observed in response to 1 and 10-μM doxo, respectively. Lastly, a further decrease up to 55% was monitored upon 20-μM doxo exposure. To note, no cytotoxicity was observed in response to vanillin alone (Figure 1B).

**Figure 2:**
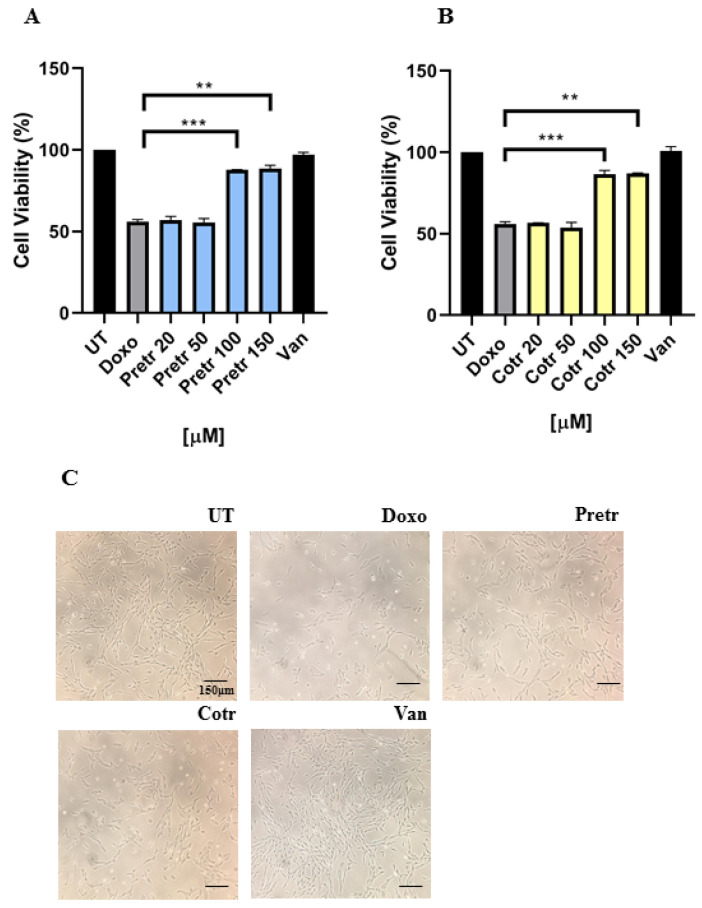
Effect of vanillin on doxo-induced cytotoxicity. Cell viability was evaluated by MTT assay in H9c2 cells exposed to doxo for 24 h, (**A**) pretreated and (**B**) co-treated with vanillin at the indicated concentrations. Data are expressed as average percentage of MTT reduction ± SD relative to untreated cells from triplicate wells from 5 separate experiments. UT—untreated cells; doxo—cells exposed to 20-μM doxo; Pretr—vanillin pretreated cells for 18 h; Cotr—vanillin cotreated cells; Van: cells exposed to 150-μM Van (**C**) Phase contrast microscopy images taken at the end of treatment time with a higher vanillin concentration. **: *p* < 0.01; ***: *p* <0.001 by one-way ANOVA. Other experimental details are described in Materials and Methods section.

Based on the above results, we decided to expose H9c2 cells to 20-μM doxo, a very high concentration, with the aim of investigating the possible protective effect of vanillin in presence of doxo-dependent incisive and forceful cell damage conditions. In details, H9c2 cells were pretreated and not with different concentrations of vanillin (20, 50, 100 and 150 μM) for 18 h and then exposed to doxo administration for additional 24 h. Even though in the pretreated samples with 20 and 50-μM vanillin doxo provoked a comparable cytotoxicity; interestingly, the usage of 100 and 150-μM vanillin significantly counteracted doxo-induced cell viability reduction (Figure 2A).

The plant-derived curcumin, whose vanillin is generated by degradation, has been demonstrated to possess similar cardioprotective feature only in pretreatment with doxo, whereas concomitant curcumin-doxo treatment potentiated the cardiotoxicity [28,29]. To figure out if vanillin had an analogous behavior when used in cotreatment with doxo, we evaluated the effects of concomitant vanillin-doxo treatment at the same time and doses already described for the pretreatment procedure. Figure 2B shows that even in cotreatment, 100 and 150-μM vanillin significantly prevented the doxo-induced toxicity in H9c2 cells, further suggesting its possible cardioprotective role against doxo exposure. Based on these results, 20-μM doxo and 100-μM vanillin doses were chosen as working concentrations for all the subsequent experiments.

Moreover, phase-contrast microscopy analysis exhibited reduction in cell amount, modification in cell morphology and cell fragments appearance in reaction to doxo treatment, whereas both co- and pre-incubation with vanillin attenuated those qualitative and quantitative alterations (Figure 2C).

Overall, these data indicate that vanillin strongly reduces doxo-induced toxicity in H9c2 cells.

### 3.2. Vanillin Prevents Doxo-Induced Cell Damage and Death in H9C2 Cells

To further investigate the protective vanillin effects in doxo-treated H9c2 cells, we also measured the release of intracellular enzymes upon cell damage [42]. Specifically, we tested the activity of lactate dehydrogenase (LDH), aspartate aminotransferase (AST) and alanine aminotransferase (ALT) in the culturing medium of cells exposed for 24 h to doxo and vanillin, both as a single agent and in combination (pre- and cotreatment). As shown in Table 1, medium from cells treated with doxo revealed a clear increase in both LDH and AST activity compared to untreated cells, thus indicating their release in response to cell membrane injury. Interestingly, the presence of vanillin, both in pre- and cotreatment conditions, completely abrogated the doxo-mediated AST increase and strongly reduced LDH activity compared to doxo alone. According to the non-functional role in the cardiac system and to its reduced expression in this body compartment, no ALT activity variations were detected in each tested sample [43].

**Figure 3:**
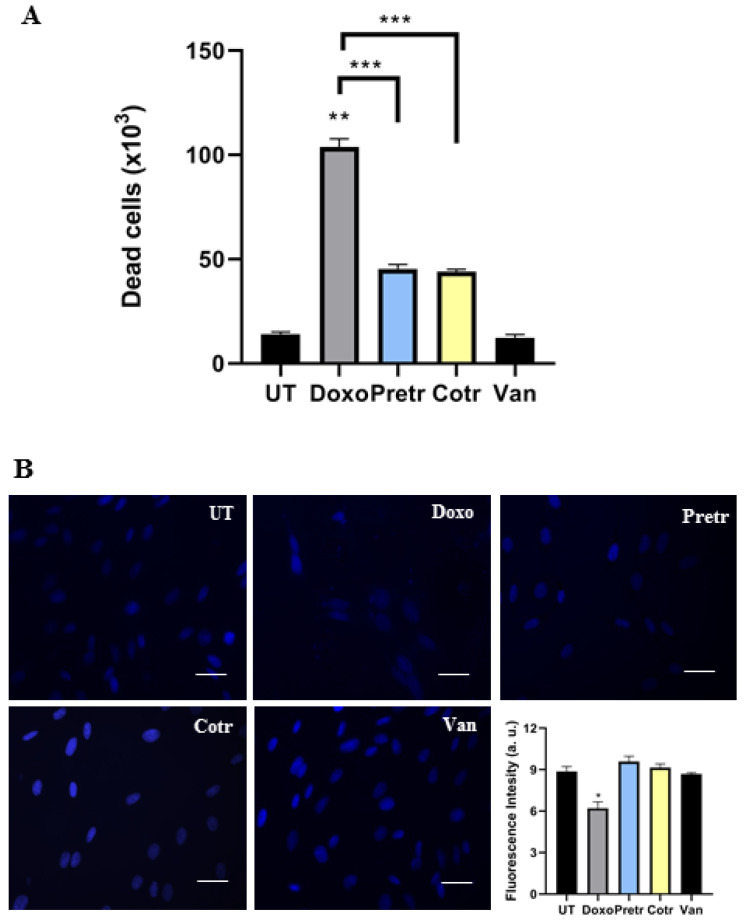
Effect of vanillin on doxo-induced cell death and nuclear alteration. (**A**) Dead cells number were evaluated by Trypan Blue assay in H9c2 cells exposed to 20 μM doxo for 24 h, pretreated (Pretr) and co-treated (Cotr) with 100-μM vanillin. UT—untreated cells; doxo—cells exposed to 20-μM doxo; Van—cells exposed to 100-μM Van. Scale bar represents 50 μm. Data represent the average of three independent experiments ± SD; (**B**) representative fluorescence images of cells stained by DAPI nuclear staining. The fluorescence intensity was estimated by Image J software. *: *p* < 0.05; **: *p* < 0.01; ***: *p* < 0.001 by two-way ANOVA. Other experimental details are described in Materials and Methods section.

In agreement with these observations, were those obtained evaluating the number of dead cells by Trypan Blu Assay. H9c2 cells were exposed for 24 h to doxo in the absence and presence of vanillin, both in pre- and cotreatment conditions, after which, blue dead cells were counted and discriminated by living cells. Figure 3A shows that in reaction to doxo administration more than 10^5^ dead cells were detected, whereas in the presence of vanillin a strong reduction in dead cells was observed (approximately 50% respect to cells treated with only doxo). Additionally, the beneficial vanillin effects were also observed by nuclear DAPI staining (Figure 3B).

According to previous findings, untreated cells were observed as round-shaped nuclei with homogeneous fluorescence intensity, whereas doxo treatment induced nuclear morphologic alterations. Conversely, in both experimental conditions in which vanillin was used in combination with doxo, nuclear integrity is maintained.

Taken together, this evidence indicates that vanillin strongly reduces doxo-induced cell damage and death in H9C2 cells.

### 3.3. Vanillin Reduces the doxo-Mediated Apoptosis in H9c2 Cells

**Figure 4:**
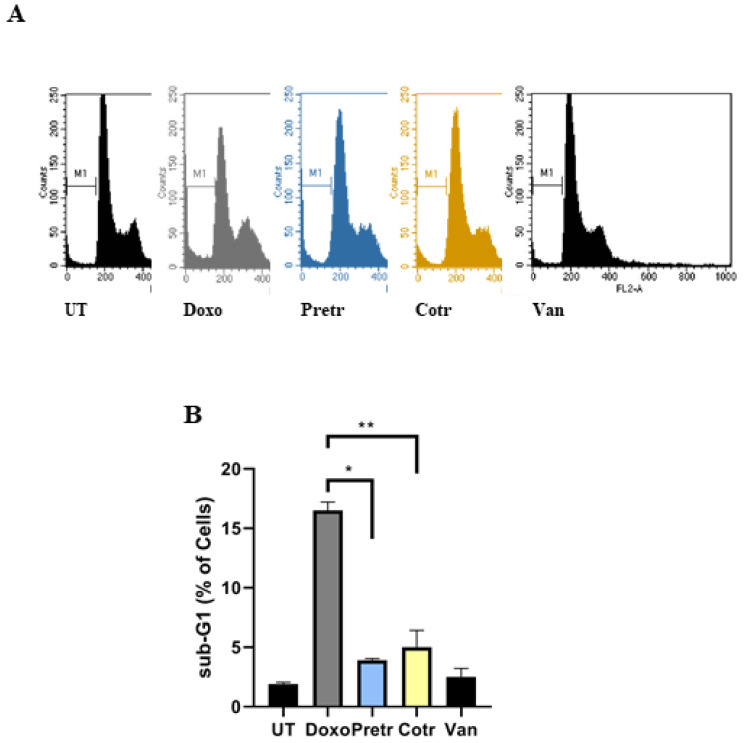
Effect of vanillin on doxo-induced sub G1-phase alteration. (**A**) Representative experiments of PI-stained H9c2 cells exposed to 20-μM doxo for 24 h, pretreated (Pretr) and co-treated (Cotr) with 100-μM vanillin. UT—untreated cells; doxo—cells exposed to 20-μM doxo; Van—cells exposed to 100-μM Van; (**B**) sub-G1 phase quantification of not less than two. Data represent the average of three independent experiments ± SD. *: *p* < 0.05; **: *p* < 0.01 by one-way ANOVA. Other experimental details are described in Materials and Methods section.

To further investigate the vanillin-mediated cell death reduction in response to doxo administration, cytofluorimetric analysis was also performed. Specifically, H9c2 cells were exposed for 24 h to doxo in the absence and presence of vanillin, both in pre- and cotreatment conditions; thereafter, PI-stained cells were analyzed by flow cytometry assay. As indicated in Figure 4, doxo exposure strongly increased the sub-G1 appearance (+17%) compared to untreated cells, meanwhile this increment was markedly reduced in presence of vanillin (4% in pretreated cells and 6% in cotreated cells).

**Figure 5:**
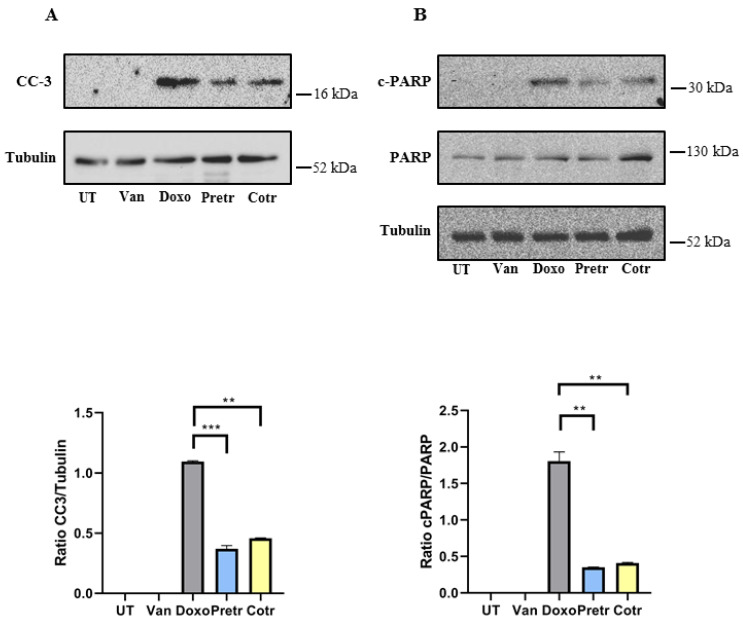
Effect of vanillin on caspase 3 and PARP1 activation. (**A**) Western blot analysis of cleaved caspase-3 (CC3) (**A**), PARP1 and cleaved PARP1 (c-PARP) (**B**) in H9c2 cells exposed to 20-μM doxo for 24 h, pretreated (Pretr) and cotreated (Cotr) with 100-μM vanillin. UT—untreated cells; doxo—cells exposed to 20-μM doxo; Van—cells exposed to 100-μM Van. Images are representative of western blotting from two different cellular extract with similar results. Data represent the average of three independent experiments ± SD. **: *p* < 0.01; ***: *p* < 0.001 by one-way ANOVA. Other experimental details are described in Materials and Methods section.

It has been reported that doxo can induce cell death by necrosis, autophagy or apoptosis in cardiomyocytes [5]. Since sub-G1 population represents the proportion of cells having undergone DNA fragmentation, one of the biochemical hallmarks of apoptosis, we also assessed the levels of some proteins involved in apoptosis in our specific conditions. To this purpose, H9c2 cells were treated in the same experimental conditions already described in Figure 4 except that, at the end of each experiments, cell extracts were prepared and analyzed by western blotting for cleaved caspase-3 (CC3), total PARP and its cleaved form (c-PARP). According to the sub-G1 data, in the samples exposed to only doxo, an increase in cleaved form of caspase-3 and PARP1 was detected. Interestingly, in the presence of vanillin, both in pre- and cotreatment, a strong reduction in the caspase-3 and PARP1 activation were observed (Figure 5). All together, these data suggest that vanillin is able to counteract the doxo-induced cell death, reducing its apoptosis induction in H9c2 cells.

### 3.4. Vanillin Prevents Doxo-Induced ROS Production and ERK1/2 Activation in H9c2 Cells

**Figure 6:**
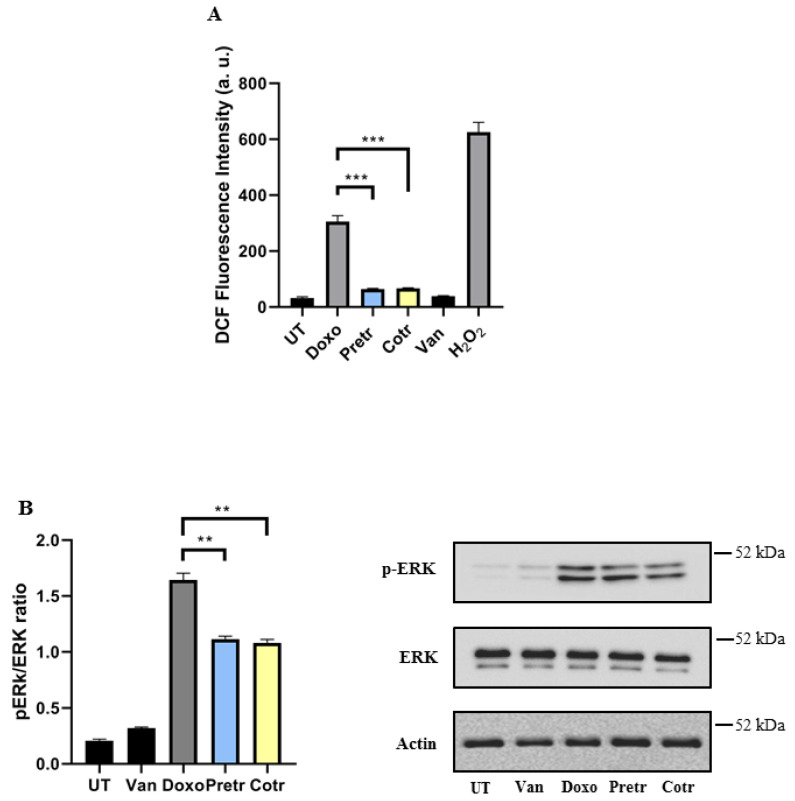
Effect of vanillin on doxo-induced ROS production and MAPK signaling. (**A**) ROS production was evaluated by DCFHDA assay in H9c2 cells exposed to 20-μM doxo for 24 h, pretreated (Pretr) and co-treated (Cotr) with 100-μM vanillin. The data are presented as mean ± SD of three replicates in five independent experiments; (**B**) western blot analysis of ERK activation. The image is representative of western blotting from two different cellular extract with similar results. UT—untreated cells; doxo—cells exposed to 20-μM doxo; Van—cells exposed to 100-μM Van; H_2_O_2_—cells treated with 1-mM H_2_O_2_; ** *p* < 0.01; *** *p* ˂ 0.001, by one-way ANOVA. Other experimental details are described in Materials and Methods section.

Oxidative stress induction and a life-threatening ROS production represent the main mechanism by which doxo generally induces cardiotoxicity [7]. In the same way, vanillin exerts its beneficial properties exactly providing an antioxidant action [37,39,44]. With the purpose of investigating the molecular mechanism associated with the beneficial effect of vanillin in preventing doxo-induced cardiotoxicity, we analyzed the ability of vanillin to affect the oxidative stress in H9c2 cells exposed to doxo. For this reason, intracellular ROS levels in H9c2 cells exposed to doxo for 24 h in the absence and presence of vanillin were measured by DCFH-DA fluorescence assay (Figure 6A). As expected, upon doxo treatment, an increase of intracellular ROS production was detected, as indicated by the increase in the DCF fluorescence. Interestingly, both the pre- and cotreatment with vanillin strongly reduced the ROS production that was similar to untreated cells.

MAPKs signaling cascade is a downstream event of ROS production, deeply involved in cell proliferation and apoptosis [45]. It has been reported that MAPK signaling pathway is involved in the doxo-induced cardiotoxicity, via phosphorylation of ERK1/2 [46,47]. For this reason, we evaluated the ERK1/2 activation in H9c2 cells exposed to doxo in the absence and presence of vanillin by western blot analysis. As displayed in Figure 6B, doxo treatment induced a strong increase in ERK1/2 phosphorylation, without significant variations in total amount. Intriguingly, in H9C2 cells pre- and cotreated with vanillin, a substantial decrease of the phospho-ERK1/2 intensity was detected.

Overall, these results suggest that the protective effect of vanillin against the doxo-induced cardiotoxicity could be mediated by its antioxidant activity, likely affecting ROS production and ROS-related pathways, such as ERK1/2.

### 3.5. Vanillin Does Not Impair Antitumor doxo Activity in U2OS Osteosarcoma Cells

**Figure 7:**
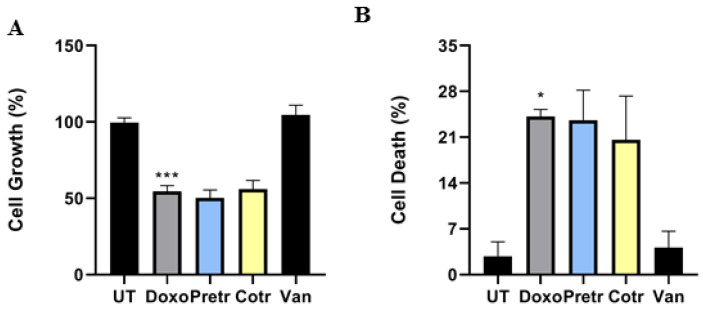
Effect of vanillin on doxo-induced antineoplastic properties in U2OS osteosarcoma cells. Number of living (**A**) and dead (**B**) U2OS cells exposed to 20-μM doxo for 24 h, pretreated (Pretr) and cotreated (Cotr) with 100-μM vanillin. UT—untreated cells; doxo—cells exposed to 20-μM doxo; Van—cells exposed to 100-μM Van; * *p* < 0.05; *** *p* ˂ 0.001 by one-way ANOVA.

In spite of the copious number of treatment-related side effects, some of them potentially lethal such as the delayed cardiotoxicity already discussed, doxo still remains the elective standard of care for several tumor types, including breast cancer and osteosarcoma [48,49]. Aiming to highlight the scientific relevance of the emerging vanillin cardioprotective skills in reaction to doxo administration, and concomitantly to exclude any possible impairment of vanillin on doxo antitumor activity, we also evaluated the effects of vanillin, doxo and their combinations in a widely used osteosarcoma cell model [50]. In detail, U2OS cells were treated in the same experimental conditions (time and doses) previously described for H9c2 cells and then evaluated for live and dead cells amount. As extensively predicable, Figure 7 shows that doxo dramatically decreased the relative number of live cells (−43%) and subsequently increased the percentage of dead cell (+22%), whereas vanillin did not affect both live and dead cells compare to control. Interestingly, although no significant additive or synergistic effects seem to become known from the evaluation of those biologic parameters, both combinatory treatments caused live cells reduction and cell death increase comparable to doxo alone.

Overall, this evidence indicates that vanillin does not impair doxo-mediated antineoplastic features in U2OS osteosarcoma cells.

## 4. Discussion

Despite of the potentially lethal delayed cardiotoxicity, doxo still remains the elective standard of care for several tumor in which no target therapy has been recognized yet [48,49]. Unfortunately, the only FDA approved cardioprotective agent (dexrazoxane) showed a limited application due to its interference with doxo efficacy, leading to secondary malignancies [9,51]. Therefore, reducing the doxo-related cardiac injuries are absolutely needed in order to extend the duration of action, and consequently to increase the doxo antitumor efficacy.

Several plant-derived polyphenols have drawn attention in pharmaceutical field thanks to their ability to reduce oxidation species synthesis and accumulation in human body [5,20,21,52,53]. Recently, it was reported that curcumin is able to ameliorate the doxo cardiotoxic effect in vitro and in vivo [22,24]. However, curcumin potential clinical application both as chemotherapeutic and cardioprotective agent remains constrained due to its poor bioavailability and low aqueous solubility [30,33]. In our previous study, we reported that curcumin and vanillin, one of the main and stable curcumin-derived degradation products, have similar effects on anti-amyloid as well as anti-AGEs formation in insulin, supporting the hypothesis that vanillin could mediate some of the biologic properties ascribed to curcumin [44]. In the present study, we demonstrated that vanillin significantly ameliorates the doxo-induced toxicity in H9c2 cells. Specifically, vanillin was able to largely restore the cell viability and damage in reaction to doxo exposure, as indicated by the reduction of the LDH and AST intracellular enzymatic release. According to these results, a strongly reduction in cell death and an attenuation of nuclear shrinkage and deformity were also induced by vanillin in presence of doxo treatment.

As largely known, doxo-induced cardiomyopathy is due to several mechanisms, including increased oxidative stress, lipid peroxidation, DNA damage, apoptosis and, more recently, autophagy [5]. However, ROS overproduction and apoptosis remain the hallmark of the doxo cardiotoxicity [3,5,6,7,8,54]. In agreement with these evidences, our results show that in the presence of vanillin a strong reduction of sub-G1 phase occurred, thus suggesting that vanillin is able to counteract the doxo-induced apoptosis. Accordingly, we observed a strong increase of cleaved caspase-3 and PARP1, two of the main markers of apoptosis, in response to doxo, that was prevented by vanillin. BAX activation has recently been indicated as rate-limiting step in doxorubicin-induced cardiomyopathy [55]. Similarly, vanillin has also been reported to modulate Bcl-2-associated X protein positively and negatively in reaction to discrete stimuli [56,57]. Therefore, we cannot exclude that other apoptotic-related proteins are involved in the vanillin-mediated cardioprotective effects in H9c2 cells. Moreover, data obtained by DCFH assay also indicate that vanillin-inhibited ROS production induced by doxo through its antioxidant activity.

The regulation of downstream pathways in response to doxo-induced ROS production could have an important role to inhibit doxo-induced toxicity. It is well-known that MAPK cascade is involved in the response to doxo-induced oxidative stress. In particular, ERKs proteins are known to be activated by doxo in H9c2 cells [46,47]. In this respect, Huang et al. has recently proved that doxo induced mitochondrial ROS release, ERK activation and HSF2-mediated AT 1 R upregulation, causing heart failure in vitro and in vivo models [58]. Based on these considerations, we detected a significant attenuation of ERK phosphorylation in presence of vanillin compared to that observed in response to doxo alone. These results suggest that vanillin could protect from doxo-induced cardiotoxicity affecting the MAPK cascade, through the modulation of the ERK activation. Although not in reaction to doxo administration, a similar vanillin-mediated signaling modulation has been reported by Dhanalakshmi and co-workers who, studying the rotenone-induced neurotoxicity in SH-SY5Y neuroblastoma cells, observed an attenuation in rotenone-induced ERK phosphorylation as a consequences of vanillin pretreatment [38]. Furthermore, even in AOM/DSS-induced colitis-associated mouse model vanillin prevents the colon cancer development weakening the NF-κB and ERK AOM/DSS-mediated activation [59]. The ability of vanillin to modulate the MAPK pathways, such as ERKs, p38 and JNKs, has also been reported in different models. Cheng and coworkers, for instance, have shown that vanillin-inhibited activator protein 1 (AP-1) in a dose-dependent manner via ERK in hepatocellular carcinoma cell line HepG2 [60].

Since curcumin showed cardioprotective properties only when used in pretreatment, but not in concomitant curcumin-doxo treatment, where instead synergistic effects were observed, we also addressed this issue analyzing both of those conditions for the entire study [28,29]. Irrespective of the administration modalities, pre- and cotreatment, we observed that vanillin attenuates doxo-induced cardiotoxicity in H9c2 cells. In reliance on this evidence, it can be deduced that vanillin is partially implicated in the curcumin-mediated cardioprotective features, but probably other curcumin-derived degradation products may be involved in. It remains unclear how the same compounds are capable of inducing opposite cardiac effects simply by changing the experimental conditions anyway.

Vanillin antioxidant and anticancer potential have extensively been described in different cancer preclinical models, such as breast, ovarian, cervical and colon cancer [37]. More recently, discrete antitumor effects have also been reported in lung, hepatocyte and neuroblastic cells. In this respect, Srinual and coworkers have established that vanillin suppresses cancer stemness phenotypes in the human non-small cell lung cancer NCI-H460 cell line through the downregulation of CD133 and ALDH1A1 and the associated transcription factors, Oct4 and Nanog [61]. Furthermore, in human hepatocyte carcinoma and neuroblastoma models the antioxidant properties are strictly related to the vanillin-mediated antineoplastic efforts, because it significantly reduces the mitochondrial membrane depolarization and ROS production, leading to apoptosis induction [62]. Taking into consideration this evidence and in light of the cardioprotective results shown in the present study, it is conceivable to develop a therapeutic regimen in which vanillin is used either as adjuvant chemotherapy agent or just heart injury defending compound. Speaking of which, a recent study reported that vanillin enhances doxo-mediated antiproliferative effects in different in vitro and in vivo breast cancer models, therefore suggesting a potential synergistic outcome [63]. Although our preliminary findings do not reveal any vanillin-mediated cytotoxic activities in U2OS osteosarcoma cells, the doxo efficacy does not seem to be hindered by the vanillin. However, further and more comprehensive studies, are required to fully define the vanillin role in osteosarcoma. It should be clarified that this interesting aspect does not represent the aim of the current study, even though it may portray our future investigation however.

In conclusion, our study indicates for the first time that vanillin is able to protect cardiomyocytes against doxo-induced cell injury. The overall data suggest that the main mechanism by which vanillin exerts its beneficial effect could be ascribed to their antioxidant properties. The inhibition of ROS production impairs the MAPK signaling thus affecting the apoptotic cell death. Despite the high doses generally employed in both in vitro and in vivo studies, vanillin shows a very wide safety profile, without any toxic effects on kidney, liver and brain tissues [37,63,64]. In agreement with the existing findings, we used vanillin in μM range whereas a doxo overdose has been applied with the purpose of exploring the vanillin-mediated protective effects in the event of deep cell damage. Even though the present study clearly demonstrates the biologic effect of vanillin in doxo-induced cardiotoxicity, the mechanisms underlying the observed protective outcome is only brushed. Moreover, extending our contribution to additional in vitro and in vivo models could further increase the clinical repercussion of these findings. In conclusions, our findings open new avenues for developing therapeutic applications for vanillin in order to counteract the anthracycline related cardiotoxicity and improving the long-term outcome of antineoplastic treatment.

## Figures and Tables

**Table 1:** Effect of vanillin on doxo-induced cellular damage. The release of AST, ALT, LDH intracellular enzymatic activities was evaluated in cultured media of H9c2 cells exposed to 20-μM doxo for 24 h, pretreated (Pretr) and cotreated (Cotr) with 100-μM vanillin. Values are means ±S.D. of triplicate samples of a typical experiment. Medium: cultured medium without cells; UT: untreated cells; doxo: cells exposed to doxo; Van: cells exposed to vanillin.

Sample	(Protein) (g/dL)	LDH (U/L)	AST (U/L)	ALT (U/L)
Medium	1.1 ± 0.05	40 ± 0.01	3 ± 0.20	6 ± 0.01
UT	1 ± 0.04	42 ± 0.03	3 ± 0.10	6 ± 0.03
Doxo	1.1 ± 0.03	212 ± 0.02	23 ± 0.05	6 ± 0.10
Pretr	1.1 ± 0.03	60 ± 0.03	3 ± 0.10	6 ± 0.20
Cotr	1.1 ± 0.04	62 ±0.05	3 ± 0.10	6 ± 0.05
Van	1 ± 0.02	45 ± 0.04	3 ± 0.20	6 ± 0.02
